# Surface relief hologram formed by selective SiO_2_ deposition on soda-lime silicate glass

**DOI:** 10.1371/journal.pone.0210340

**Published:** 2019-01-24

**Authors:** Daisuke Sakai, Kenji Harada, Hiroyuki Shibata, Keiga Kawaguchi, Junji Nishii

**Affiliations:** 1 Faculty of Engineering, School of Regional Innovation and Social Design Engineering, Kitami Institute of Technology, Kitami, Hokkaido, Japan; 2 Faculty of Engineering, School of Earth, Energy and Environmental Engineering, Kitami Institute of Technology, Kitami, Hokkaido, Japan; 3 Research Institute for Electronic Science, Hokkaido University, Sapporo, Hokkaido, Japan; Washington State University, UNITED STATES

## Abstract

We propose the fabrication of surface relief holograms via selective SiO_2_ deposition on soda-lime silicate glass substrates. Initially, the original hologram was recorded on an azobenzene photosensitive polymer film coated on the soda-lime silicate glass by irradiation with a conventional continuous wave Ar^+^ laser with a wavelength of 514.5 nm. The hologram was transferred to the soda-lime silicate glass surface via a corona discharge treatment as an index modulation hologram, which was created by partial substitution of protons for sodium ions during the corona discharge treatment in air. After the corona discharge treatment, the polymer film was removed from the substrate. The diffraction efficiency of the index hologram on the soda-lime silicate glass was estimated to be 5.8 × 10^−2^% at a wavelength of 532 nm. Finally, the glass substrate was subjected to corona discharge treatment in air with vaporized cyclic siloxane. A surface relief hologram with the diffraction efficiency of 2.3% was successfully fabricated on the soda-lime silicate glass.

## Introduction

Holograms are used to record secure information, display three dimensional images, and create holographic optical elements. Since the early days of holograms, various materials have been studied for recording holographs, such as silver halide photosensitive materials, photopolymers, and dichromated gelatins [1–3]. Rewritable, post-treatment-free, and polarization recording functional holographic recording materials have been reported, the most notable of which are azobenzene containing polymers [4,5].

Photosensitive materials can record holograms simply via irradiation with a laser beam at their absorption wavelength. Some organic polymer materials exhibit high photosensitivity, but they are not very stable under humid conditions, at high temperatures, and under intense ultra-violet light. Oxide glass, meanwhile, is attracting attention as a holographic recording material owing to its stable and long-term recording capabilities. However, with this material it is difficult to record holograms using conventional holographic recording techniques because of its high transmittance and low photosensitivity. New recording techniques are therefore being examined, such as ultrashort pulsed-laser illumination using a femto-second laser [6]. Laser-induced backside etching using a nanosecond-pulsed laser scan has also been reported for inscribing the grating on the silica glass [7]. Moreover, Nastas et al. reported a holographic recording technique for chalcogenide glass using laser irradiation in conjunction with a corona discharge [8,9].

We studied a technique for transferring a hologram from an azobenzene polymer onto a soda-lime silicate (SLS) glass substrate using a corona discharge treatment (CDT) [10,11]. The azobenzene polymer hologram was recorded using a conventional continuous wave (CW) laser, such as an Ar^+^ laser or a Nd:YVO_4_ laser. The diffraction efficiency of the index modulation hologram transferred to the SLS glass was as high as 10^−2^% [12]. In our most recent study, we reported a microfabrication method to selectively deposit SiO_2_ onto the electrically nanoimprinted SLS glass using CDT in air with added vaporized cyclic siloxane [13]. However, the fabrication costs and durability of nanoimprint molds are insufficient for practical use. This paper reports the fabrication of surface relief holograms with high diffraction efficiencies via selective deposition of SiO_2_ on SLS glass using CDT.

## Materials and methods

**Fig 1:**
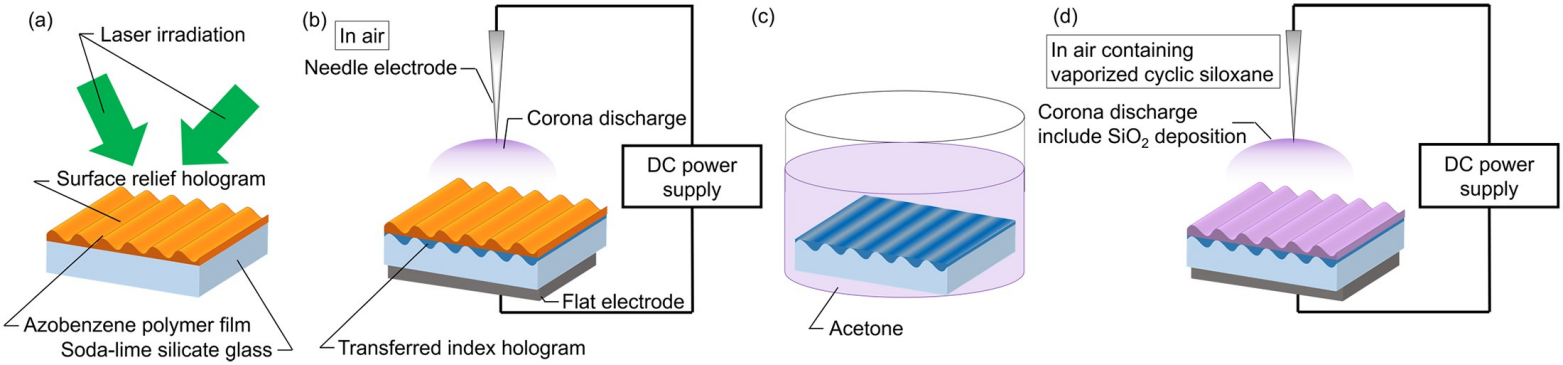
Schematic drawing of the experimental procedure. (a) Holographic recording. (b) Corona discharge. (c) Removal of the polymer. (d) Corona discharge deposition.

The experimental procedures are shown in Fig 1. First, a surface relief hologram was recorded on a photosensitive polymer film (Fig 1a). A SLS glass substrate with dimensions of 25 × 25 × 1 mm^3^ was used. A side-chain type azobenzene polymer (poly-orange tom-1; Trichemical Laboratories Inc.) dissolved in cyclohexanone at 10 wt% was spincoated onto the substrate. The film was baked on a hot plate heated at 150 °C for 10 min. The film thickness was approximately 400 nm. The surface relief hologram was recorded by exposure using the two-beam interference method. A circular polarized continuous wave Ar^+^ laser (Stabilite 2017; wavelength (λ): 514.5 nm; Spectra-Physics Inc.) was used as the light source.

Second, the CDT was performed to transfer the hologram to the substrate, as shown in Fig 1b. The CDT setup was placed in a maffle furnace (FO200; Yamato Scientific Co., Ltd.). A nickel-coated steel needle and a stainless-steel plate were used as the anode and cathode electrodes, respectively. The hologram-recorded sample was set on the plate electrode and subjected to positive corona discharge in the air at 136 °C for 2 h. The corona plasma was generated by applying a direct current (DC) voltage of +6 kV to the needle electrode, which was fixed 10 mm above the sample surface. The azopolymer film was then removed from the sample (Fig 1c). The polymer film was dissolved and cleaned by ultra-sonic cleaning using acetone as a solvent and was then subsequently annealed.

Finally, the SiO_2_ was deposited on the hologram-transferred SLS glass substrate using the CDT. The CDT setup was the same with that shown in Fig 1b. Vaporized cyclic siloxane, which was generated from the adhesive of heat resistant tape (Nitoflon adhesive tape No. 903UL; vaporization temperature of the adhesive: 180°C; Nitto Denko corp.) by heating it at 200 °C in a furnace, was added to the air.

The diffraction efficiencies of the recorded holograms were measured using a He–Ne laser (λ: 633 nm) for the azopolymer and Nd:YVO_4_ laser (λ: 532 nm) for the SLS glass substrate. The surface profiles were observed using an atomic force microscope (AFM; Nanocute; SII Nanotechnology Inc.).

## Results and discussion

**Fig 2:**
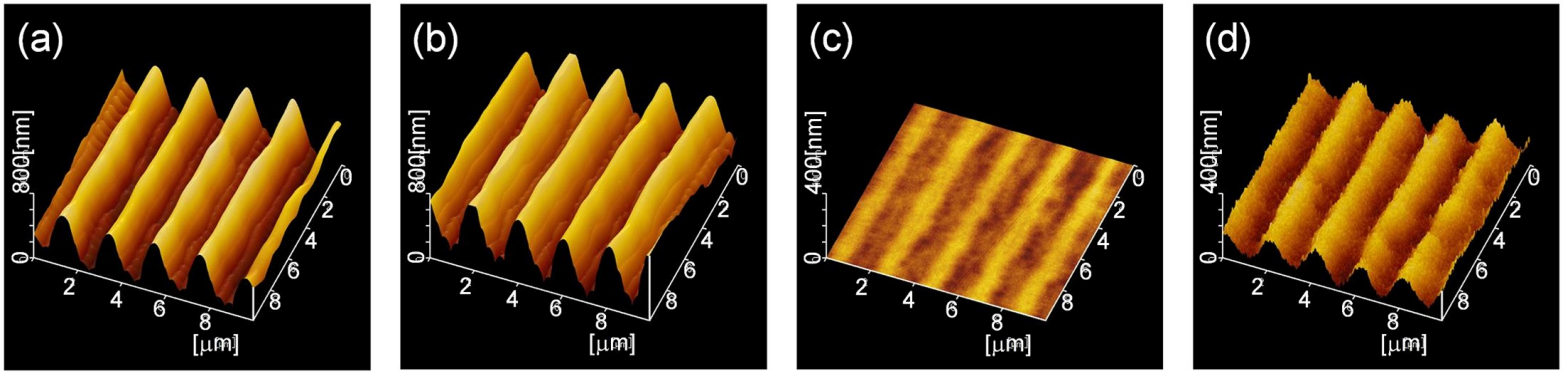
Surface profile of experimental sample. Azobenzene polymer (a) after holographic recording and (b) after corona discharge. Glass substrate (c) after removal of polymer and (d) after corona discharge deposition.

Fig 2 shows the topographical AFM images obtained at each experimental step. A photo-induced surface relief hologram with a grating constant of approximately 2 μm was formed on the azopolymer film as a structural template, as shown in Fig 2a. Fig 2b shows the surface profile on the azopolymer film after CDT. The diffraction efficiency increased from 20 to 27% with the depth amplification of the hologram formed by the CDT, which is in agreement with results in previous reports [14,15]. A hologram with an identical grating constant was transferred onto the SLS glass as the index modulation hologram with a shallow surface relief (Fig 2c) [12]. Fig 2d indicates the surface profile of the hologram on the SLS glass substrate after corona discharge deposition (CDD) of the SiO_2_. The diffraction efficiency increased to 2.3% even though the efficiency was 5.8 × 10^−2^% before CDD. The index modulation hologram gradually became a surface relief hologram consisting of selectively deposited SiO_2_, which caused the amplification of the diffraction efficiency.

**Fig 3:**
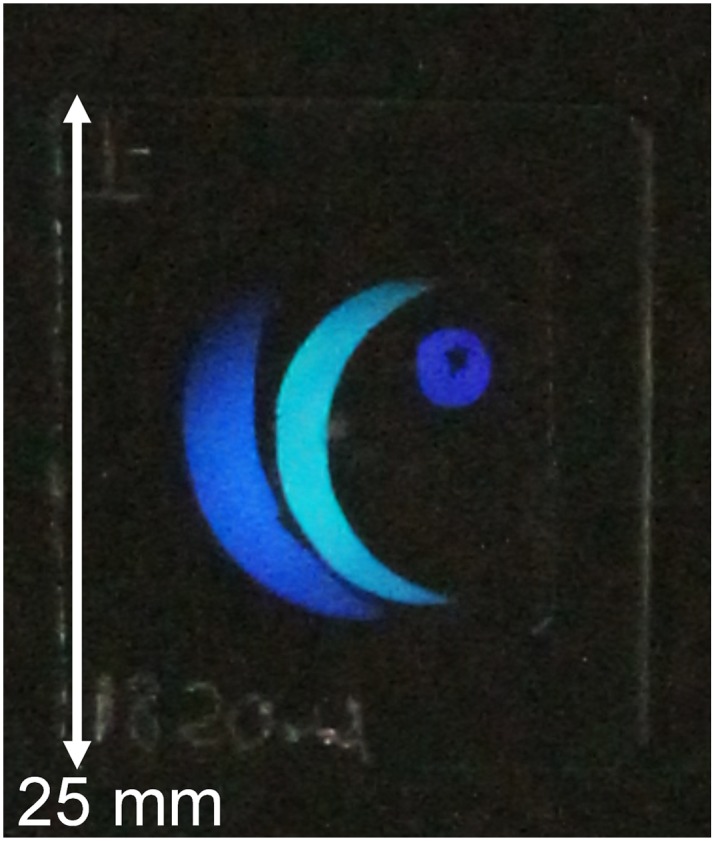
Photograph of the reconstructed image hologram consisting of SiO_2_ on soda-lime silicate glass.

Fig 3 shows the image hologram fabricated on the SLS glass after the SiO_2_ deposition for 50 min. The index modulation hologram on the transparent glass substrate was barely visible because of the low diffraction efficiency. However, the hologram became visible on the sample surface after the SiO_2_ deposition in line with an increase in the diffraction efficiency. The size of the treated area was 20 mm in diameter. The grating period was kept constant throughout our experiments. Thus, a surface relief hologram consisting of SiO_2_ was successfully fabricated on transparent SLS glass based on the recording with a conventional CW laser.

**Fig 4:**
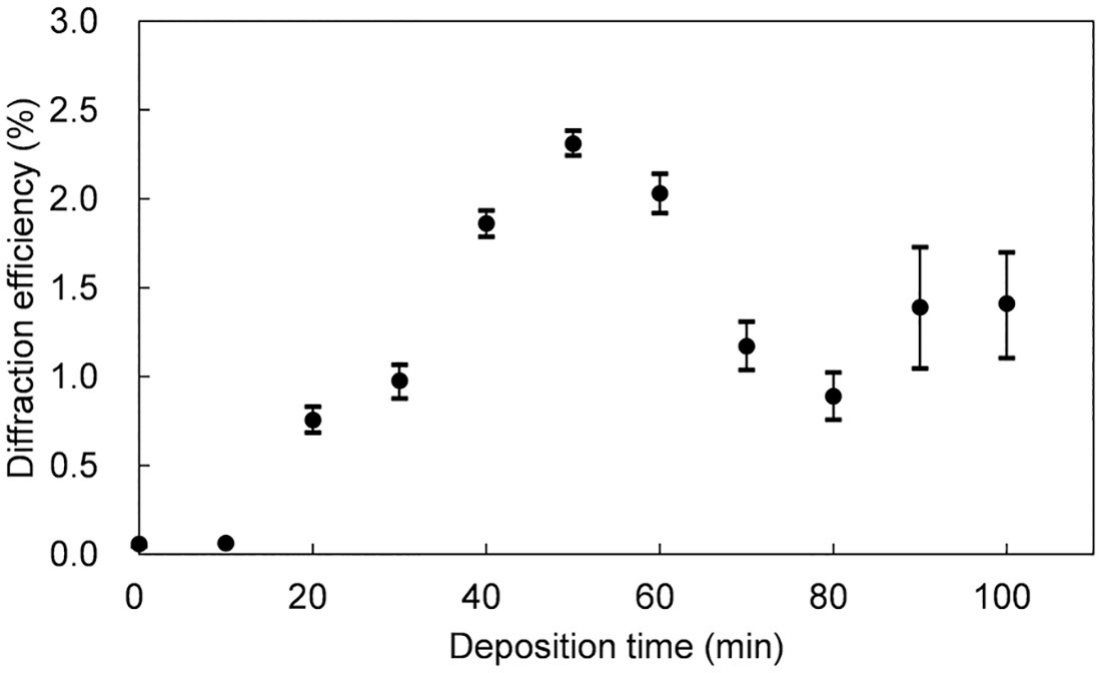
Diffraction efficiency as a function of the deposition time.

Fig 4 shows the relationship between the diffraction efficiency and the duration of the CDD. A CDD duration of 50 min caused the diffraction efficiency to increase 40 times (to its maximum); for longer durations the efficiency was shown to be lower, at ca. 1.5%.

A similar selective SiO_2_ deposition has previously been reported on electrically nanoimprinted SLS glass [13]. The difference in ion conductivities between the area in which sodium ions remained and the depleted area on the SLS glass surface was the origin of the selective SiO_2_ deposition, as the SiO_2_ deposition was suppressed in the sodium ion depleted area. The sodium depleted area was formed at the contact area of the electro-conductive mold to which a positive DC voltage was applied; oxygen deficient defects thus formed accompanied by the vaporization of oxygen molecules [16]. Conversely, in this study, the hologram was transferred onto the SLS glass surface via an azobenzene polymer grating using CDT. During the CDT, the sodium ions in the glass are substituted by protons supplied from the corona plasma based on the azobenzene grating pattern [17,18]. If the conductivities of the injected protons lower than that of the sodium ions, then it is expected that the deposition of SiO_2_ onto the proton injected area will be inhibited.

**Fig 5:**
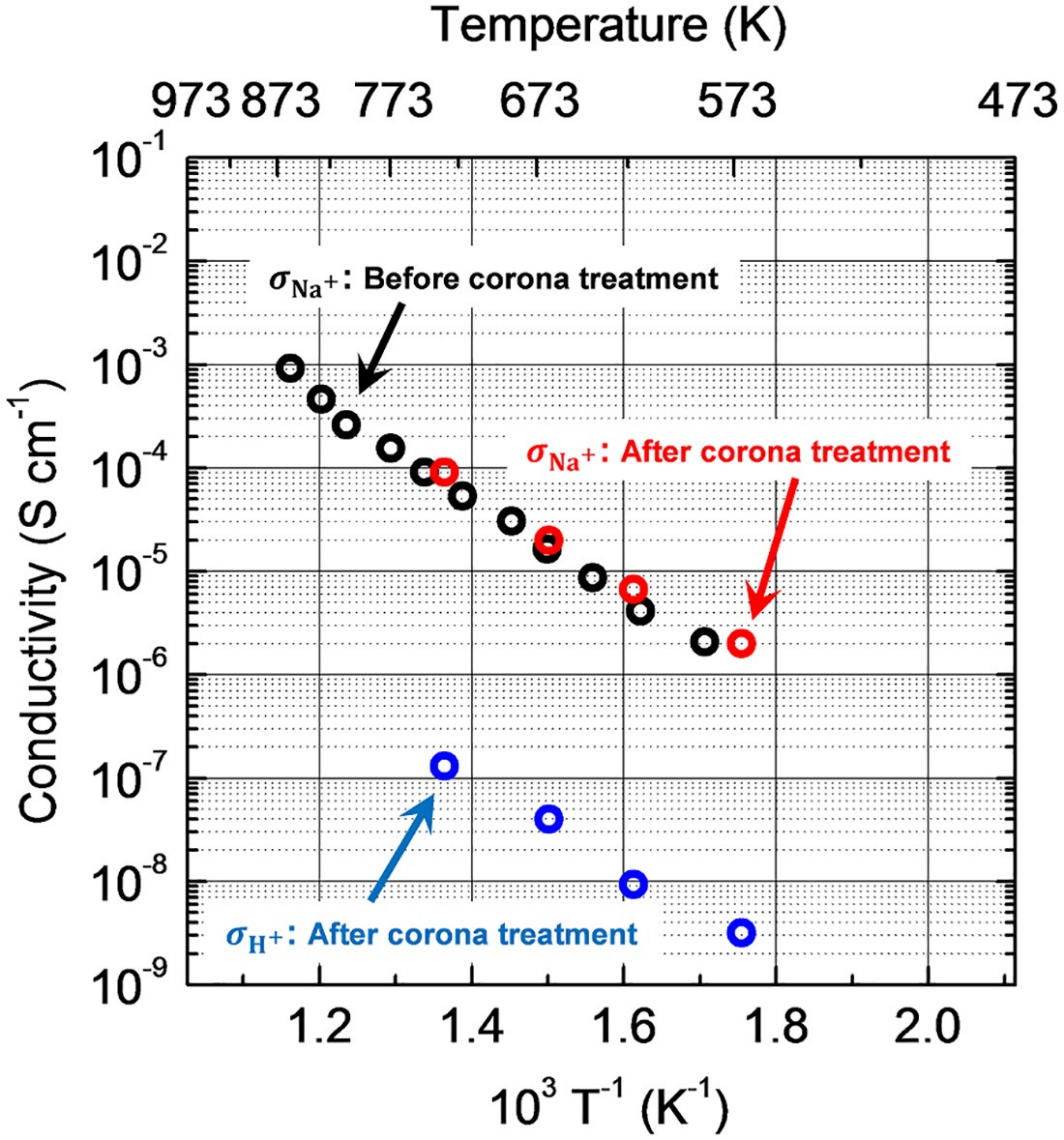
Ion conductivities of sodium ions and protons in the SLS glass before and after CDT. Ion conductivities are indicated as σ. No proton conduction was visible before the CDT.

We determined the ion conductivity of the corona discharge treated soda-lime silicate glass using the four-electrode alternating-current impedance method. The terminals of the electrodes were connected to an impedance/gain phase analyzer (4194A; Hewlett Packard Inc.). The temperature dependence of the impedances of the soda-lime silicate glass with or without CDT were measured between 1.0 × 10^2^ and 1.5 × 10^7^ Hz in a 20% H_2_−80% N_2_ atmosphere. Fig 5 shows Arrhenius plots of the ion conductivities estimated from the impedance analysis. The SLS glass before CDT exhibited a Nyquist diagram attributed to sodium ion conduction. In contrast, an additional resistance component was detected for the glass after CDT, which can likely be ascribed to proton conduction from protons injected into the sodium ion site by the CDT. The conductivities of the sodium ions in the SLS glass with or without the corona discharge were almost the same. The conductivity of protons injected into the SLS glass was three orders of magnitude lower than that of sodium ions, i.e., a periodic pattern of different conductivities was formed in the glass that matched the grating period of the hologram throughout the CDT. This significant decrease in the conductivity is in agreement with similar trends found in previous studies regarding thermal polling on SLS glass [19,20]. Therefore, this result suggests the origin of the deposition selectivity is the large difference in ion conductivities between the sodium ions and the injected protons in the soda-lime silicate glass.

**Fig 6:**
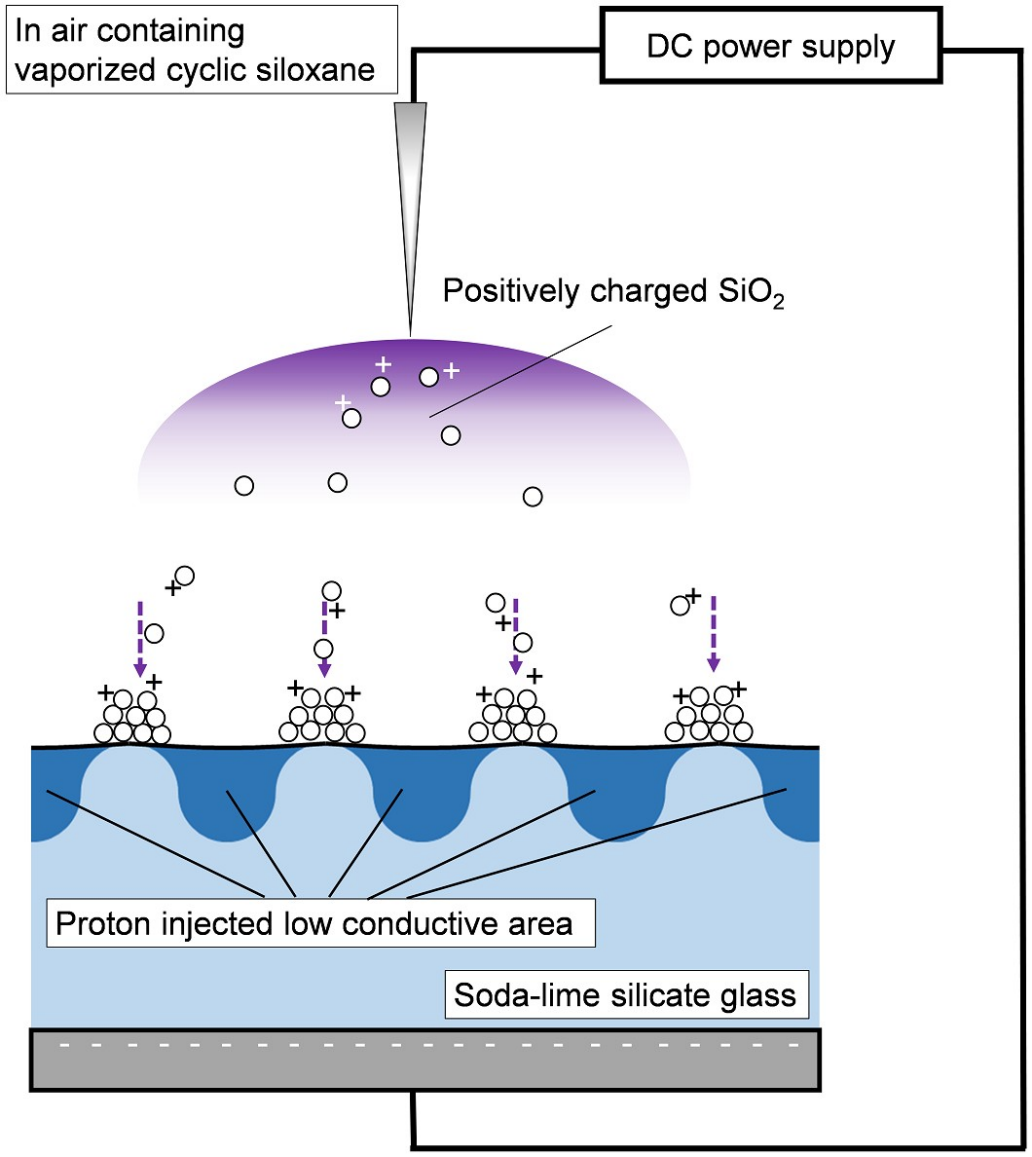
Schematic image of the formation of a surface relief hologram consisting of SiO_2_ during CDD.

Fig 6 is a schematic diagram illustrating the fabrication of the surface relief hologram via SiO_2_ using CDD. Vaporized cyclic siloxane in the air is decomposed by positive corona plasma around the tip of a sharp needle electrode; positively charged SiO_2_ is subsequently generated. The positively charged SiO_2_ flies selectively toward higher conductivity areas on the glass, such as where sodium ions are present. This selective deposition resulted in the formation of a surface relief structure with the same spatial frequency as that of the hologram. Hence, the diffraction efficiency of the hologram increased with the structural height, which depended on the CDD time. The diffraction efficiency of the CDD hologram was decreased at the surrounding area, which was attributed to the low substitution rate from sodium ions to protons.

Under our experimental conditions the diffraction efficiency saturated before finally decreasing after reaching its maximum for a CDD duration of 50 min. This result means the deposition selectivity was degraded for longer CDD durations because the development of the structural height stopped at 50 min. The periodically present sodium ions then gradually migrated from the anode side toward the cathode side as current carriers in the SLS glass during continued CDD. When the difference in the ion conductivities became negligibly small, the whole surface on the hologram was covered with a uniformly deposited SiO_2_ film after prolonged CDD. The important point is the formation of fine pattern with high-resistivity contrast on the glass surface. The modification of glass composition is effective to attain a high resistance by the substitution of alkali metal ion to proton. Another point is the resistivity of photosensitive polymer. The proton injected into polymer must path through only its thinner area to the glass surface. Therefore, the proton diffusivity in polymer resist is also important to attain the high aspect ratio.

## Conclusions

We proposed a fabrication method for surface relief holograms via selective deposition of SiO_2_ on SLS glass using CDT. We thus realized a hologram recorded using the two-beam interference method with an Ar^+^ laser; transparent soda-lime silicate glass was used as the substrate. An original hologram was recorded on a photo-sensitive azobenzene polymer film on the glass substrate. The hologram was transferred onto the glass substrate using CDT. A transferred index modulation hologram was formed via the periodic substitution of protons for sodium ions in the glass. After the azobenzene polymer was removed, the glass onto which the hologram had been transferred was again subjected to corona discharge in air containing vaporized cyclic siloxane.

Positively charged SiO_2_, which was generated by the decomposition of vaporized cyclic siloxane, was selectively deposited on the SLS glass surface, and thus the surface relief hologram was formed. The deposition selectivity originated from large difference in conductivity owing to the index hologram on the surface of the SLS glass. Using four-electrode alternating-current impedance analysis we confirmed that the conductivity of the corona-injected protons in the SLS glass was three orders of magnitude lower than the sodium ion conductivity.

The advantage of our method is the stable and large-area recording of hologram on the stable oxide glass using a conventional CW laser. This is applicable to larger area using other type of electrode, such as wire. Our method is expected to be highly cost effective compared with other method using ultrashort pulsed-lasers or less stable non-oxide glasses. These are advantages in the industry applications compared with other methods. Furthermore, a higher diffraction efficiency could be achieved by optimizing the experimental conditions and maintaining the deposition selectivity; the method could be applied to holographic diffractive optical elements and stable holographic memory.

## Supporting information

S1 Supporting Information Numerical data set of Figs 4 and 5. (PDF)
