# Supplementary material for: Surface relief hologram formed by selective SiO2 deposition on soda-lime silicate glass
Source: PLoS One. 2019 Jan 24;14(1):e0210340. doi: 10.1371/journal.pone.0210340 (PMC6345422; doi:10.1371/journal.pone.0210340)
Supplement: S1 Supporting Information — (PDF) [file pone.0210340.s001.pdf]

S1 Supporting Information

Title: Surface relief hologram formed by selective SiO_2 deposition on soda-lime silicate glass

Author: Daisuke Sakai, Kenji Harada, Hiroyuki Shibata, Keiga Kawaguchi, and Junji Nishii

S1 Table. Numerical data set of the diffraction efficiencies, which build Fig 4.

<table>
<tr><th>Deposition time (min)</th><th colspan="3">Diffraction efficiency (%)</th></tr>
<tr><td rowspan="2">0</td><td rowspan="2">0.058</td><td>min</td><td>0.043</td></tr>
<tr><td>max</td><td>0.063</td></tr>
<tr><td rowspan="2">10</td><td rowspan="2">0.063</td><td>min</td><td>0.060</td></tr>
<tr><td>max</td><td>0.065</td></tr>
<tr><td rowspan="2">20</td><td rowspan="2">0.755</td><td>min</td><td>0.684</td></tr>
<tr><td>max</td><td>0.831</td></tr>
<tr><td rowspan="2">30</td><td rowspan="2">0.976</td><td>min</td><td>0.875</td></tr>
<tr><td>max</td><td>1.066</td></tr>
<tr><td rowspan="2">40</td><td rowspan="2">1.860</td><td>min</td><td>1.787</td></tr>
<tr><td>max</td><td>1.934</td></tr>
<tr><td rowspan="2">50</td><td rowspan="2">2.310</td><td>min</td><td>2.243</td></tr>
<tr><td>max</td><td>2.382</td></tr>
<tr><td rowspan="2">60</td><td rowspan="2">2.030</td><td>min</td><td>1.919</td></tr>
<tr><td>max</td><td>2.140</td></tr>
<tr><td rowspan="2">70</td><td rowspan="2">1.170</td><td>min</td><td>1.037</td></tr>
<tr><td>max</td><td>1.309</td></tr>
<tr><td rowspan="2">80</td><td rowspan="2">0.888</td><td>min</td><td>0.757</td></tr>
<tr><td>max</td><td>1.022</td></tr>
<tr><td rowspan="2">90</td><td rowspan="2">1.390</td><td>min</td><td>1.044</td></tr>
<tr><td>max</td><td>1.728</td></tr>
<tr><td rowspan="2">100</td><td rowspan="2">1.410</td><td>min</td><td>1.103</td></tr>
<tr><td>max</td><td>1.699</td></tr>
</table>

S2 Table. Numerical data set of the ion conductivities, which build Fig 5.

A. Before corona treatment

Temperature		Ion conductivity (Scm^{-1})
1000/T	T(K)	Na^+
1.706	586.233	2.090E-06
1.622	616.668	4.126E-06
1.559	641.313	8.651E-06
1.499	666.963	1.642E-05
1.453	688.464	3.037E-05
1.388	720.585	5.323E-05
1.338	747.372	9.144E-05
1.294	772.990	1.557E-04
1.235	809.497	2.606E-04
1.203	831.430	4.597E-04
1.162	860.636	9.226E-04

B. After corona treatment

Temperature		Ion conductivity (Scm^{-1})	
1000/T	T(K)	Na^+	H^+
1.754	570.000	1.996E-06	3.158E-09
1.613	620.000	6.655E-06	9.287E-09
1.502	666.000	1.996E-05	4.014E-08
1.364	733.000	9.075E-05	1.298E-07
